# Peripapillary RNFL/vessel density ratio in patients with type2 diabetes without clinical diabetic retinopathy

**DOI:** 10.1038/s41598-022-13567-6

**Published:** 2022-06-08

**Authors:** Min-Woo Lee, Jong-Wook Lee, Kook-Hyung Lee, Young-Hoon Lee, Jung-Yeul Kim

**Affiliations:** 1 Department of Ophthalmology, Konyang University College of Medicine, Daejeon, Republic of Korea; 2 Department of Ophthalmology, Chungnam National University College of Medicine, #640 Daesa-dong, Jung-gu, Daejeon, 301-721 Republic of Korea; 3 1.0 Eye clinic, Daejeon, Republic of Korea

**Keywords:** Medical research, Eye diseases

## Abstract

To identify how diabetic retinal neurodegeneration (DRN) and microvascular impairment are affected differently by various factors in type 2 diabetes (T2DM) patients without diabetic retinopathy via the ratio of RNFL thickness/vessel density (RNFL/VD) ratio. In this retrospective cross-sectional study, subjects were divided into two groups: controls (control group) and patients with T2DM (DM group). The RNFL thickness, VD, and RNDL/VD ratio were compared between two groups, and correlation analyses were performed to identify the relationship between the RNFL/VD ratio and various factors. A total of 411 eyes were enrolled: 195 eyes in the control group and 216 eyes in the DM group. The mean RNFL thickness was 95.9 ± 8.6 and 93.7 ± 8.7 μm (P = 0.016), the VD was 18.2 ± 0.7 and 17.6 ± 1.1 mm^−1^ (P < 0.001), and the RNFL/VD ratio was 5.11 ± 0.47 and 5.22 ± 0.53 (P = 0.033) in the control group and DM group, respectively. In the DM group, age (coefficient = − 0.139, P = 0.041), axial length (coefficient = 0.163, P = 0.017), and T2DM duration (coefficient = − 0.180, P = 0.008) were significantly correlated with the RNFL/VD ratio. The RNFL/VD ratio of T2DM patients was higher than that of normal control, which would indicate that the impairment of microvasculature precedes DRN. Additionally, age and T2DM duration were negatively correlated with the RNFL/VD ratio, which suggests that inner retinal damage by DRN becomes more prominent over time than microvascular impairment in T2DM.

## Introduction

Type 2 diabetes (T2DM) is a global health problem causing severe mortality and morbidity, and its prevalence has been increasing from 108 million in 1980 to 422 million in 2014^1,2^. It has also been estimated that there were 451 million people with diabetes worldwide in 2017, with cases expected to increase to 693 million by 2045^3^. This increase in the prevalence of T2DM naturally leads to an increase in the prevalence of diabetic retinopathy (DR), one of the most common microvascular complications of T2DM^4^. DR, the leading cause of blindness in the working population globally, can cause diabetic macular edema, tractional retinal detachment, and neovascular glaucoma, which would result in permanent visual loss without appropriate management^5^. Therefore, T2DM patients with DR changes including retinal hemorrhage and hard exudates should be observed carefully for prevention of DR-related complications.

Meanwhile, anatomical and functional damage of the retina has been reported in T2DM patients without any DR change these days^6–8^. Diabetic retinal neurodegeneration (DRN) is representative retinal damage preceding DR change in T2DM patients, which causes rapid inner retinal reduction including peripapillary retinal nerve fiber layer (pRNFL) and ganglion cell layer^9–11^. Additionally, the impairment of peripapillary and macular microvasculature could occur before a definite DR change in T2DM patients^7,8,12,13^. They may lead to various impairments of visual function in T2DM patients such as loss of dark adaptation and contrast sensitivity, color vision disturbance, abnormal microperimetry, and low visual acuity^14–16^. These two types of retinal damage, DRN and microvasculature impairment, are thought to be related to each other by neurovascular coupling. Although previous studies reported the relationship between them, the mechanism of the association between them has not been fully elucidated^8,12^. Analysis of the sequence of these two damages can help understand the mechanisms underlying their association, and also help establish a potential treatment strategy for retinal damages occurring before clinical DR changes in the future.

In this study, we tried to identify the sequence of DRN and microvascular impairment by analyzing pRNFL thickness, vessel density (VD), and the ratio of pRNFL thickness/VD, and to understand their mechanisms in more detail by analyzing their associations with various factors in T2DM patients without clinical DR.

## Methods

### Patients

This retrospective, cross-sectional study adhered to the tenets of the Declaration of Helsinki and was approved by the Institutional Review Board of Chungnam National University Hospital, Daejeon, Republic of Korea. We reviewed the charts of patients with T2DM who visited the retinal clinic of Chungnam National University Hospital for DR checkups from March 2017 to June 2021. The requirement for obtaining informed consent was waived due to the retrospective nature of the study. We recorded detailed histories and measurements of best-corrected visual acuity (BCVA), intraocular pressure, spherical equivalent, and axial length. Subjects were divided into two groups: controls (control group) and patients with T2DM (DM group). The control group included patients without any systemic disease including T2DM, and diagnosed with a unilateral epiretinal membrane, macular hole, retinal detachment, or intraocular lens dislocation; all of the fellow eyes without any ophthalmic disease. The data was collected from these healthy fellow eyes. The exclusion criteria consisted of a history of systemic disease other than T2DM, glaucoma, optic nerve disorder, intraocular pressure ≥ 21 mmHg, axial length ≥ 26.0 mm, or any other optic nerve or retinal dysfunction. We also excluded patients with clinical evidence of DR on color fundus photo and fluorescein angiography, such as retinal hemorrhage or microaneurysm. If both eyes met the inclusion criteria in one person, one eye was randomly selected.

### OCT and OCTA measurements

Spectral domain-OCT (SD-OCT) examinations were performed using a Cirrus HD-OCT 5000 (Carl Zeiss Meditec, Dublin, CA, USA) with an optic disc cube scan. A 200 × 200 scan optic cube scanning protocol was used to image the optic disc and pRNFL over a 6 × 6 mm region of the optic nerve head.

**Figure 1:**
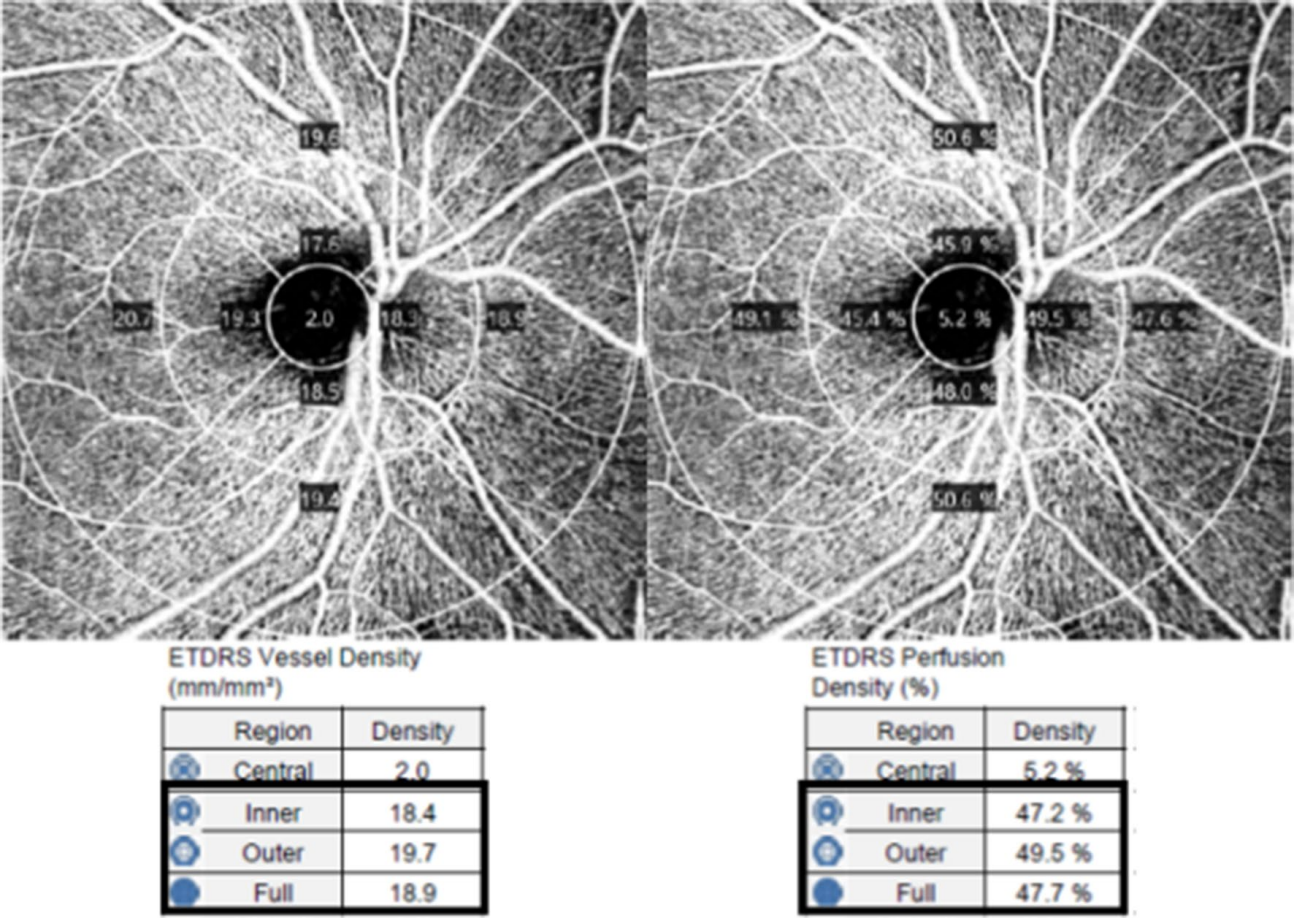
A representative 6 × 6 mm optical coherence tomography angiography centered on the optic disc. The en face image of the superficial capillary plexus overlaid with the Early Treatment of Diabetic Retinopathy Study grid. The black boxes show the automatic quantitative measurements for an average vessel density and perfusion density of the inner ring, outer ring, and full area.

OCTA examinations were performed using a Cirrus HD-OCT 5000 with AngioPlex software (version 10.0; Carl Zeiss Meditec), with a wavelength of 840 nm taking 68,000 A-scans per second. The device ensures sensitivity and accuracy by incorporating the optical microangiography (OMAG) algorithm and retinal tracking technology. We obtained an optic disc centered scan using a 6 × 6 mm pattern mode, which contains an approximate area of 6 × 6 mm size over the optic nerve head. All scans were analyzed using en face OCTA images generated automatically by the OMAG algorithm used in AngioPlex software as a previous study^13^. VD was defined as the total length of perfused vasculature per unit area in the region of measurement, and perfusion density (PD) was defined as the total area of perfused vasculature per unit area in a region of measurement. The AngioPlex software quantified VD and PD via the Early Treatment of Diabetic Retinopathy Study subfields (Fig. 1). We analyzed the peripapillary VD and PD in the superficial capillary plexus of the quadrants of the inner and outer rings, and 6 mm full area. All images were checked and verified by two blinded observers (L.M.W. and J.Y.K.), and any images exhibiting fixation or centration loss, segmentation errors, motion artifacts, or signal strengths < 9 were excluded.

### Statistical analysis

Demographic characteristics and ocular parameters were compared via an independent t-test and a chi-square test. Pearson correlation analyses were performed to identify the relationship between the mean RNFL thickness/VD (RNFL/VD) and various factors; we analyzed the VD of the outer ring to exclude the optic disc area. All statistical analyses were performed using SPSS software (version 18.0; IBM Corp., Armonk, NY, USA).

## Results

### Demographics

A total of 411 eyes were enrolled: 195 eyes in the control group and 216 eyes in the DM group. The mean age was 58.4 ± 11.7 and 60.5 ± 9.4 years in the control group and the DM group, respectively, which was not significantly different (P = 0.071) (Table 1). The mean BCVA of each group was − 0.01 ± 0.06 and 0.01 ± 0.07, respectively (P < 0.001). Other characteristics including sex, laterality, spherical equivalent, intraocular pressure, and axial length were not significantly different between the two groups.

**Table 1:** Demographics and clinical characteristics.

	Control group (n = 195)	DM group (n = 216)	P value
Age (mean ± SD, year)	58.4 ± 11.7	60.5 ± 9.4	0.071
Sex (%, male)	87	101	0.368
Laterality (%,right)	94	111	0.293
Duration of diabetes (mean ± SD, year)	n/a	7.4 ± 6.2	n/a
HbA1c (mean ± SD, %)	n/a	6.9 ± 0.9	n/a
BCVA (mean ± SD, logMAR)	− 0.01 ± 0.06	0.01 ± 0.07	** < 0.001**
Spherical equivalent (mean ± SD, diopter)	− 0.52 ± 2.04	− 0.23 ± 1.70	0.122
Intraocular pressure (mean ± SD, mmHg)	14.7 ± 2.7	15.3 ± 2.8	0.070
Axial length (mean ± SD, mm)	23.9 ± 1.1	23.8 ± 0.9	0.412
Central macular thickness (mean ± SD, μm)	253.5 ± 18.5	252.4 ± 28.1	0.614

Significant values are in bold.

*BCVA* best-corrected visual acuity.

### pRNFL thickness and OCTA parameters

The mean pRNFL thickness was 95.9 ± 8.6 and 93.7 ± 8.7 μm in the control group and DM group, respectively, and they were significantly different (P = 0.016) (Table 2). In sectoral thickness, superior and inferior sectors of the DM group were significantly thinner than those of the control group (P = 0.015 and P = 0.049, respectively).

**Table 2:** Peripapillary retinal nerve fiber layer thickness and parameters of optical coherence tomography angiography.

	Control group	DM group	P value
**pRNFL thickness (mean ± SD, μm)**			
Mean	95.9 ± 8.6	93.7 ± 8.7	**0.016**
Superior	120.8 ± 20.2	116.8 ± 17.3	**0.015**
Temporal	72.4 ± 10.5	71.0 ± 12.9	0.291
Inferior	123.3 ± 15.1	120.1 ± 19.8	**0.049**
Nasal	68.4 ± 9.1	70.1 ± 13.8	0.179
**OCTA parameters**			
VD full area (mean ± SD, mm^−1^)	18.2 ± 0.7	17.6 ± 1.1	** < 0.001**
VD outer ring (mean ± SD, mm^−1^)	18.8 ± 0.7	18.2 ± 1.3	** < 0.001**
VD inner ring (mean ± SD, mm^−1^)	17.6 ± 1.3	17.2 ± 1.6	**0.015**
PD full area (mean ± SD, %)	46.5 ± 3.1	44.6 ± 4.6	** < 0.001**
PD outer ring (mean ± SD, %)	47.6 ± 1.7	46.1 ± 4.2	** < 0.001**
PD inner ring (mean ± SD, %)	46.1 ± 3.5	45.2 ± 4.3	**0.020**

*pRNFL* peripapillary retinal nerve fiber layer, *OCTA* optical coherence tomography angiography, *VD* vessel density, *PD* perfusion density.

Significant values are in bold.

In OCTA parameters, the VD of the full area was 18.2 ± 0.7 and 17.6 ± 1.1 mm^−1^ in the control group and DM group, respectively, which was significantly different (P < 0.001). The VDs of outer and inner rings were also significantly different between the two groups (P < 0.001 and P = 0.015, respectively). The PD of the full area was 46.5 ± 3.1 and 44.6 ± 4.6% in each group, respectively, and they were significantly different (P < 0.001). The PDs of outer and inner rings were significantly different between the two groups, similar to the VD results.

### pRNFL thickness/OCTA ratio of each group

**Figure 2:**
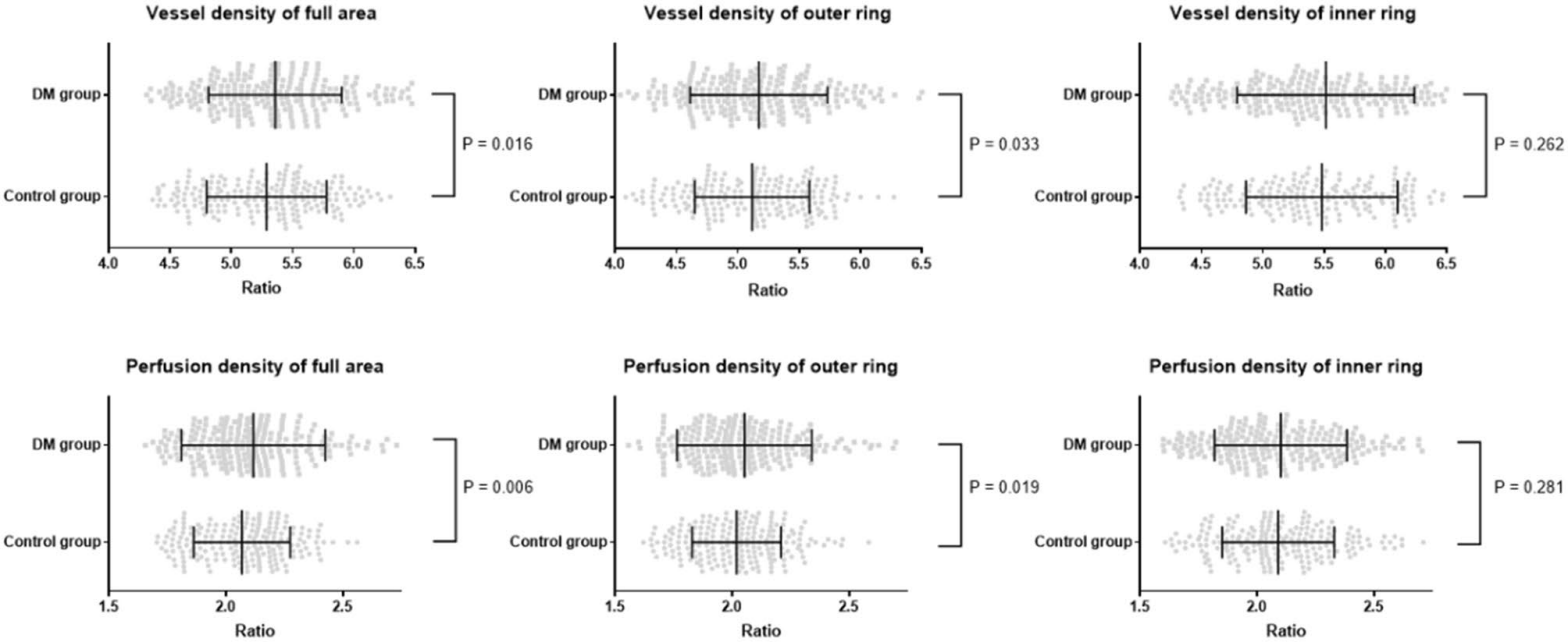
Scatter plots showing the mean RNFL thickness/parameters of optical coherence tomography angiography ratio in each group.

The RNFL/VD full area ratio was 5.28 ± 0.49 and 5.40 ± 0.51 in the control and DM groups, respectively, which was significantly different (P = 0.016) (Table 3). The RNFL/VD outer ring in each group was 5.11 ± 0.47 and 5.22 ± 0.53, respectively, and they were also significantly different (P = 0.033). The RNFL/VD inner ring ratio of the control group was lower than that of the DM group, but it was not statistically significant (P = 0.262). The RNFL/PDs ratio showed similar results to the ratio of VDs (Fig. 2).

**Table 3:** The mean retinal nerve fiber layer thickness/vessel density ratio in each group.

	Control group	DM group	P value
RNFL/VD full area	5.28 ± 0.49	5.40 ± 0.51	**0.016**
RNFL/VD outer ring	5.11 ± 0.47	5.22 ± 0.53	**0.033**
RNFL/VD inner ring	5.48 ± 0.62	5.57 ± 0.70	0.262
RNFL/PD full area	2.07 ± 0.21	2.16 ± 0.43	**0.006**
RNFL/PD outer ring	2.01 ± 0.19	2.07 ± 0.28	**0.019**
RNFL/PD inner ring	2.09 ± 0.24	2.12 ± 0.28	0.281

Significant values are in bold.

*RNFL* retinal nerve fiber layer, *VD* vessel density, *PD* perfusion density.

In the DM group, patients with T2DM duration ≤ 5 years showed significantly higher RNFL /VD full area and RNFL/VD outer ring ratios than patients with T2DM duration > 5 years (5.48 ± 0.59 vs 5.33 ± 0.42, P = 0.035; 5.31 ± 0.63 vs 5.14 ± 0.41, P = 0.023, respectively).

### Correlation analyses between RNFL/VD outer ring ratio and various factors

In the DM group, age (coefficient = − 0.139, P = 0.041), axial length (coefficient = 0.163, P = 0.017), and T2DM duration (coefficient = -0.180, P = 0.008) were significantly correlated with the RNFL/VD ratio (Table 4). In the control group, none of the factors were significantly correlated with the RNFL/VD ratio.

**Table 4:** Correlation analyses between the mean retinal nerve fiber layer thickness/vessel density ratio and various factors.

	Control group		DM group	
	Coefficient	P value	Coefficient	P value
Age	− 0.026	0.713	− 0.139	**0.041**
Sex	− 0.023	0.752	− 0.045	0.512
Laterality	0.052	0.474	0.057	0.404
Duration of diabetes	n/a	n/a	− 0.180	**0.008**
HbA1c	n/a	n/a	− 0.018	0.816
BCVA	0.138	0.054	0.094	0.167
Spherical equivalent	0.116	0.108	0.031	0.650
Intraocular pressure	− 0.103	0.152	0.023	0.733
Axial length	− 0.066	0.362	0.163	**0.017**

Significant values are in bold.

*BCVA* best-corrected visual acuity.

## Discussion

In this study, we investigated pRNFL thickness, peripapillary VD, and their ratio in T2DM patients without clinical DR to understand the relationship between DRN and microvascular impairment in more detail, and found that pRNFL thickness and VD were significantly reduced in the DM group, and the RNFL/VD ratio was significantly increased in the DM group compared to the control group. Patients with relatively short T2DM duration showed a higher RNFL/VD ratio than patients with a longer T2DM duration, which implies that prominent microvascular damage may precede inner retinal reduction by DRN. Additionally, this ratio was significantly related to age, T2DM duration, and axial length in the DM group, whereas none of the factors showed a significant relationship in the control group.

Previous studies reported a significant progressive loss of the RNFL and the ganglion cell layer in patients with DM^6,17^. Our study also showed a significantly thinner pRNFL in T2DM patients. T2DM induces activation of microglial cells which migrate to the subretinal space and release cytokines contributing to neuronal cell death^9,18^. Such neuronal loss of the retina could result in a reduction of the pRNFL thickness in T2DM patients even though they do not show any DR changes such as retinal hemorrhage, hard exudates, or cotton wool spots. Additionally, Vujosevic et al.^8^ reported a significant decrease in peripapillary VD in the DM groups compared to the control group, which was similar to the results of our study. The accumulation of glutamate and the loss of neuroprotective factors trigger vascular endothelial growth factor activation, and neuronal death and glial dysfunction lead to vasoregression, which could result in early microvascular impairment in T2DM^9,10^. These retinal damages are related to various visual functions^7,12,13,19,20^. Therefore, the status of microvasculature and pRNFL thickness should be observed carefully in T2DM patients, even if they do not show signs of clinical DR.

We tried to infer the sequence of the DRN and microvascular impairment by analyzing the RNFL/VD ratio and found that the RNFL/VD ratio was significantly higher in the DM group than in the control group. In the situation of a decrease in both pRNFL thickness and VD by T2DM, the higher ratio would mean a more prominent impairment of VD than that of pRNFL. DRN and microvascular impairment in T2DM are closely related to each other and share similar mechanisms triggered by hyperglycemia, which we mentioned above. However, the temporal relationship has yet to be clarified. The higher value of the RNFL/VD ratio in T2DM patients without clinical DR implies that prominent microvascular damage may precede inner retinal reduction by DRN. Although DRN would occur before DR-related microvascular damages including microaneurysm or retinal hemorrhage, the impairment of peripapillary microvasculature may occur before a definite inner retinal reduction.

Patients with relatively short T2DM duration showed a higher RNFL/VD ratio than patients with a longer T2DM duration. After microvascular impairment was preceded in the early stages of T2DM, inner retinal reduction due to DRN would be accompanied over time, which resulted in a lowered ratio once again. Taken together, these results support the hypothesis that the impairment of peripapillary microvasculature precedes DRN. However, there could be some bias coming from the exclusion of clinical DR. Patients with relatively longer T2DM duration are more likely to experience DR change, along with greater microvascular impairment. As we excluded such patients with DR change, there was a possibility that only those with relatively minimal microvascular damage were enrolled in the DM group with relatively longer T2DM duration. Further longitudinal studies are needed to confirm this hypothesis.

Age was negatively correlated with the RNFL/VD ratio in T2DM patients. Although it is controversial, Lee et al.^18^ reported that age significantly influenced changes in the pRNFL thickness over time in patients with T2DM. However, previous studies did not show a significant relationship between age and retinal microvasculature in T2DM^12,13,21^. In older T2DM patients, inner retinal damage by DRN seemed to be more prominent than microvascular impairment. Additionally, T2DM duration was negatively related to the RNFL/VD ratio, which would mean that the reduction in pRNFL thickness was more prominent than the microvascular impairment with increasing T2DM duration. T2DM duration is a well-known factor associated with both DRN and impairment of retinal microvasculature^6,7,11–13,17,18^. As mentioned above, inner retinal damage by DRN seemed to appear gradually and severely over time after microvascular damage which occurred in the early stage of T2DM. In other words, damage to the inner retina would become more prominent than damage to the retinal microvasculature over time in T2DM.

Axial length was positively correlated with the RNFL/VD ratio in the DM group. Bundenz et al.^22^ reported that for every 1 mm increase in axial length, the mean pRNFL was measured as being thinner by approximately 2.2 μm in normal subjects. Other previous studies have also reported a negative association between pRNFL thickness and axial length^23,24^. Meanwhile, He et al.^25^ reported that radial peripapillary complex VD showed a significantly negative correlation with axial length. Lee et al.^13^ also reported that axial length was a significant factor associated with peripapillary VD in T2DM patients. As such, both the pRNFL thickness and peripapillary VD had a negative relationship with axial length, and the VD seemed to be more affected than the pRNFL thickness by the axial length in T2DM from the results of a positive correlation between axial length and the RNFL/VD ratio. In T2DM, the retinal microvasculature would be influenced more prominently than the structure of the inner retinal layer by axial elongation. However, we did not enroll patients with axial length ≥ 26.0 mm, and did not perform the corrections of the OCTA image scale according to the axial length, which may contribute to a slight image scale difference among individuals. As such, analyses involving the axial length would be somewhat limited.

This study had several limitations. First, the retrospective nature of the study inevitably introduced some selection bias. Prospective longitudinal studies are needed to confirm our hypothesis. Second, since eyes with axial length ≥ 26.0 mm were excluded, additional studies including high myopia are needed to analyze the relationship more precisely between axial length and other parameters. The strength of this study was that we enrolled a relatively large number of patients, and enrolled OCTA images with signal strength > 8 allowing for accurate analyses. Additionally, this is the first study to infer the temporal relation of DRN and retinal microvasculature impairment.

In conclusion, the RNFL/VD ratio of T2DM patients was higher than that of normal controls, and patients with relatively short T2DM duration showed a higher ratio than patients with a longer T2DM duration. These would indicate that the impairment of peripapillary microvasculature precedes DRN and that the peripapillary microvasculature is affected more prominently than the inner retina at the early stages of T2DM. Additionally, age and T2DM duration were negatively correlated with the RNFL/VD ratio, from which it could be inferred that inner retinal damage by DRN becomes more prominent than microvascular impairment over time in T2DM. These results are expected to be helpful for physicians interpreting changes in the inner retina and OCTA parameters in T2DM patients and may also be useful for a better understanding of the pathogenesis of the early stages of the disease to find out more efficient preventive strategies for retinal damage in T2DM.
